# Supplementary material for: NanoPlex: a universal strategy for fluorescence microscopy multiplexing using nanobodies with erasable signals
Source: Nat Commun. 2024 Oct 10;15:8771. doi: 10.1038/s41467-024-53030-w (PMC11479620; doi:10.1038/s41467-024-53030-w)
Supplement: Supplementary file 2 — Reporting Summary [file 41467_2024_53030_MOESM2_ESM.pdf]

nature portfolio

Corresponding author(s): Felipe Opazo

Last updated by author(s): Sep 27, 2024

Reporting Summary

Nature Portfolio wishes to improve the reproducibility of the work that we publish. This form provides structure for consistency and transparency in reporting. For further information on Nature Portfolio policies, see our Editorial Policies and the Editorial Policy Checklist.

Statistics

For all statistical analyses, confirm that the following items are present in the figure legend, table legend, main text, or Methods section.

n/a	Confirmed	
[ ]	[x]	The exact sample size (*n*) for each experimental group/condition, given as a discrete number and unit of measurement
[ ]	[x]	A statement on whether measurements were taken from distinct samples or whether the same sample was measured repeatedly
[ ]	[x]	The statistical test(s) used AND whether they are one- or two-sided<br>*Only common tests should be described solely by name; describe more complex techniques in the Methods section.*
[ ]	[x]	A description of all covariates tested
[ ]	[x]	A description of any assumptions or corrections, such as tests of normality and adjustment for multiple comparisons
[ ]	[x]	A full description of the statistical parameters including central tendency (e.g. means) or other basic estimates (e.g. regression coefficient) AND variation (e.g. standard deviation) or associated estimates of uncertainty (e.g. confidence intervals)
[ ]	[x]	For null hypothesis testing, the test statistic (e.g. *F*, *t*, *r*) with confidence intervals, effect sizes, degrees of freedom and *P* value noted<br>*Give P values as exact values whenever suitable.*
[x]	[ ]	For Bayesian analysis, information on the choice of priors and Markov chain Monte Carlo settings
[x]	[ ]	For hierarchical and complex designs, identification of the appropriate level for tests and full reporting of outcomes
[ ]	[x]	Estimates of effect sizes (e.g. Cohen's *d*, Pearson's *r*), indicating how they were calculated

Our web collection on statistics for biologists contains articles on many of the points above.

Software and code

Policy information about availability of computer code

Data collection	Proprietary software for controlling microscopes were needed for imaging acquisition.
Data analysis	Data analysis is explained in the Methods section, principally, it was done using imageJ existing plug-ins. (also clarified in Methods)

For manuscripts utilizing custom algorithms or software that are central to the research but not yet described in published literature, software must be made available to editors and reviewers. We strongly encourage code deposition in a community repository (e.g. GitHub). See the Nature Portfolio guidelines for submitting code & software for further information.

Data

Policy information about availability of data

All manuscripts must include a data availability statement. This statement should provide the following information, where applicable:

- Accession codes, unique identifiers, or web links for publicly available datasets
- A description of any restrictions on data availability
- For clinical datasets or third party data, please ensure that the statement adheres to our policy

The data that support the findings of this study are available from the corresponding author upon request.

Human research participants

Policy information about studies involving human research participants and Sex and Gender in Research.

Reporting on sex and gender	No humans or human materials were used in this study.
Population characteristics	No humans or human materials were used in this study.
Recruitment	No humans or human materials were used in this study.
Ethics oversight	No humans or human materials were used in this study.

Note that full information on the approval of the study protocol must also be provided in the manuscript.

Field-specific reporting

Please select the one below that is the best fit for your research. If you are not sure, read the appropriate sections before making your selection.

- [x] Life sciences
- [ ] Behavioural & social sciences
- [ ] Ecological, evolutionary & environmental sciences

For a reference copy of the document with all sections, see nature.com/documents/nr-reporting-summary-flat.pdf

Life sciences study design

All studies must disclose on these points even when the disclosure is negative.

Sample size	No sample-size calculation was performed nor necesary
Data exclusions	No data was excluded of any analysis performed.
Replication	The experiments that required a replication to prove the effect was not chance, were done as independent experimental repetition and after analysis their variance and standard deviation were calculated
Randomization	This point is not relevant for our study. We designed, characterized and optimized protocols in vitro
Blinding	This point is not relevant for our study. We designed, characterized and optimized protocols in vitro

Reporting for specific materials, systems and methods

We require information from authors about some types of materials, experimental systems and methods used in many studies. Here, indicate whether each material, system or method listed is relevant to your study. If you are not sure if a list item applies to your research, read the appropriate section before selecting a response.

Materials & experimental systems

n/a	Involved in the study	
[ ]	[x]	Antibodies
[ ]	[x]	Eukaryotic cell lines
[x]	[ ]	Palaeontology and archaeology
[ ]	[x]	Animals and other organisms
[x]	[ ]	Clinical data
[x]	[ ]	Dual use research of concern

Methods

n/a	Involved in the study	
[x]	[ ]	ChIP-seq
[x]	[ ]	Flow cytometry
[x]	[ ]	MRI-based neuroimaging

Antibodies

Antibodies used	The full list of antibodies used in this study are all in tables including catalog number, provider and RRID if exist.
Validation	We relied on validation performed by commercial provider. However most antibodies used for immunofluorescence reveal characteristic structures that are clearly recognizably, thus confirming their specificity in this study.

Eukaryotic cell lines

Policy information about cell lines and Sex and Gender in Research

Cell line source(s)	The COS-7 were purchased from the German Collection for Microorganisms and Cell Cultures (DSMZ), Braunschweig, Germany. U2OS-NUP96-GFP are derived from Ries Lab, cells were characterized in this paper: https://www.nature.com/articles/s41592-019-0574-9
Authentication	COS-7 cells were not authenticated in the lab, U2OS cells have a clear GFP signal in the nuclear membrane, making easy their identification under the microscopy
Mycoplasma contamination	all cell lines were subjected to a check using PCR for presence of Mycoplasma contamination.
Commonly misidentified lines (See ICLAC register)	So far neither COS-7 nor U2OS cells were reported by the ICLAC to be misidentified.

Animals and other research organisms

Policy information about studies involving animals; ARRIVE guidelines recommended for reporting animal research, and Sex and Gender in Research

Laboratory animals	Wistar RjHan:WI (RRID:RGD_13508588) rats (Rattus norvegicus)
Wild animals	No
Reporting on sex	No selection on Sex was performed
Field-collected samples	Pregnant Rats were kept in one Opti-Rat IVC cage under a 12h light/dark cycle with food and water available ad libitum. Rats were euthanized with CO2 followed by cervical dislocation.
Ethics oversight	Animals were obtained from the University Medical Center Göttingen and handled according to the specifications of the University of Göttingen and the local authority, the State of Lower Saxony (Landesamt für Verbraucherschutz, LAVES, Germany). The local authority (Lower Saxony State Office for Consumer Protection and Food Safety) approved the animal experiments (license number: T09/08)

Note that full information on the approval of the study protocol must also be provided in the manuscript.
